# End-member modelling as a tool for climate reconstruction—An Eastern Mediterranean case study

**DOI:** 10.1371/journal.pone.0185136

**Published:** 2017-09-21

**Authors:** Sarah Beuscher, Stefan Krüger, Werner Ehrmann, Gerhard Schmiedl, Yvonne Milker, Helge Arz, Hartmut Schulz

**Affiliations:** 1 Universität Leipzig, Institut für Geophysik und Geologie, Leipzig, Germany; 2 Universität Hamburg, Centrum für Erdsystemforschung und Nachhaltigkeit, Hamburg, Germany; 3 Leibniz-Institut für Ostseeforschung, Rostock, Germany; 4 Universität Tübingen, Fachbereich Geowissenschaften, Tübingen, Germany; Universita degli Studi di Urbino Carlo Bo, ITALY

## Abstract

The Eastern Mediterranean Sea is a sink for terrigenous sediments from North Africa, Europe and Asia Minor. Its sediments therefore provide valuable information on the climate dynamics in the source areas and the associated transport processes. We present a high-resolution dataset of sediment core M40/4_SL71, which was collected SW of Crete and spans the last ca. 180 kyr. We analysed the clay mineral composition, the grain size distribution within the silt fraction, and the abundance of major and trace elements. We tested the potential of end-member modelling on these sedimentological datasets as a tool for reconstructing the climate variability in the source regions and the associated detrital input. For each dataset, we modelled three end members. All end members were assigned to a specific provenance and sedimentary process. In total, three end members were related to the Saharan dust input, and five were related to the fluvial sediment input. One end member was strongly associated with the sapropel layers. The Saharan dust end members of the grain size and clay mineral datasets generally suggest enhanced dust export into the Eastern Mediterranean Sea during the dry phases with short-term increases during Heinrich events. During the African Humid Periods, dust export was reduced but may not have completely ceased. The loading patterns of two fluvial end members show a strong relationship with the Northern Hemisphere insolation, and all fluvial end members document enhanced input during the African Humid Periods. The sapropel end member most likely reflects the fixation of redox-sensitive elements within the anoxic sapropel layers. Our results exemplify that end-member modelling is a valuable tool for interpreting extensive and multidisciplinary datasets.

## Introduction

**Fig 1:**
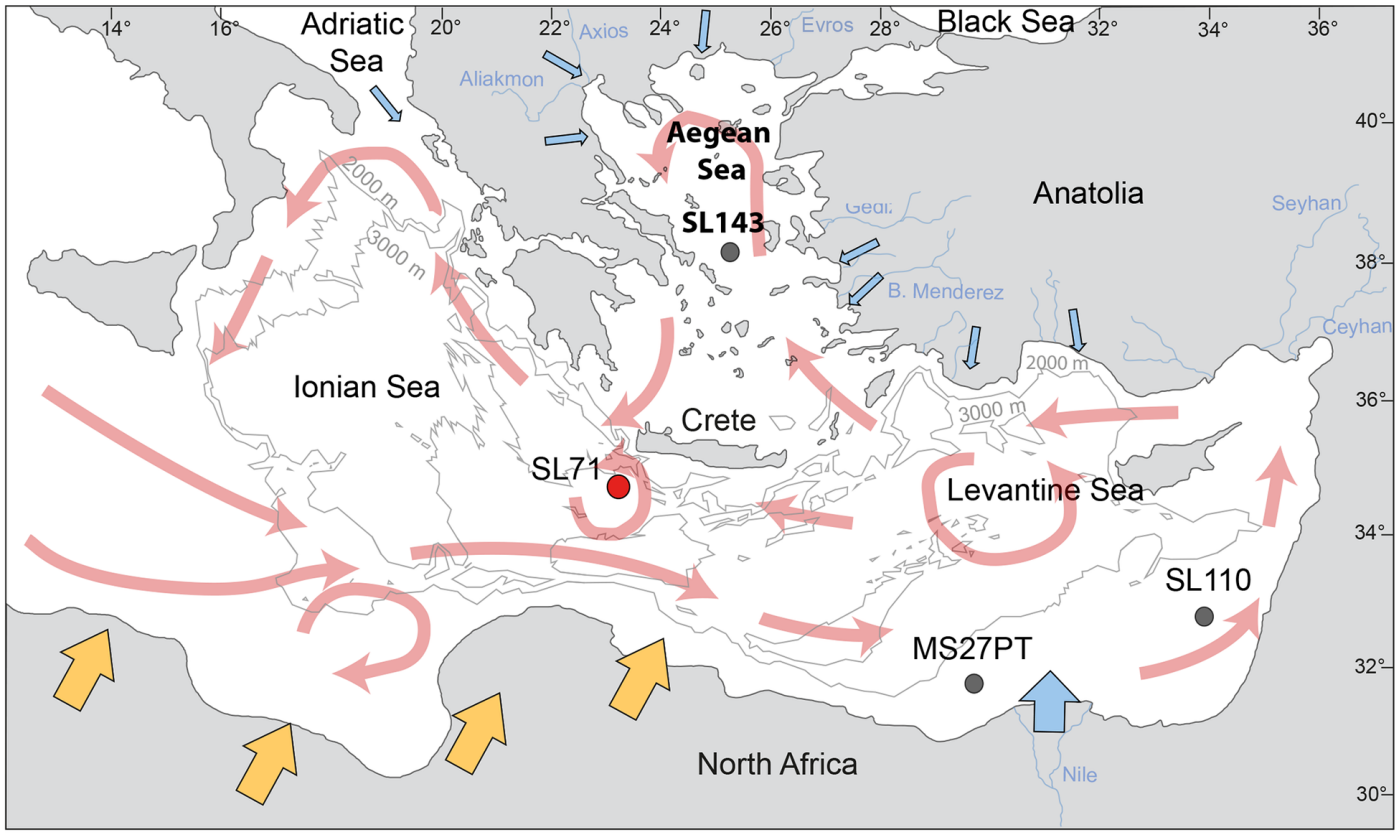
Map of the Eastern Mediterranean Sea with the location of the investigated sediment core M40/4_SL71 investigated in this study, redrafted after [1]. The general surface circulation pattern in the Eastern Mediterranean Sea is marked in red, after [2, 3]. The 2000 m and 3000 m isobaths are included in light grey. Blue arrows indicate the input of riverine sediment, orange arrows indicate the input of Saharan dust. The positions of the sediment cores that are mentioned in the text, M51/3_SL143, M51/3_SL110, are also indicated.

The Eastern Mediterranean Sea is a semi-enclosed ocean basin between Africa, Asia and Europe (Fig 1, redrafted after [1]). This basin is situated between the warm and dry climate of northern Africa to the south and the temperate and humid climate of Europe to the north. This basin acts as a sediment sink for the erosion products delivered from the adjacent landmasses by fluvial and aeolian processes and pathways. Ocean currents [2, 3] mix and distribute the sediments within the basin. The marine deposits provide information about the environmental changes on the adjacent landmasses and their reaction to past climate changes.

Data of the grain size, clay mineral, geochemical and isotopic compositions have been widely used to reconstruct the provenance and transport processes for the Eastern Mediterranean Sea sediments. This information can be further used to decipher the archived climatic signals [4–10].

The Nile River is quantitatively the primary source for riverine sediment in the Eastern Mediterranean Sea [11], supplying the southeastern Levantine Sea. Several Anatolian rivers discharge their sediment loads into the northern Levantine Sea. The Aegean Sea receives sediment via the Black Sea outflow and by the rivers draining southeast Europe and western Turkey (compilation in [12]). Finally, the Po River feeds a large amount of sediment into the Adriatic Sea [13]. Dust-bearing winds reach the Eastern Mediterranean Sea from Europe, the Near East and the Sahara. The Saharan desert is quantitatively the primary source for dust in the Eastern Mediterranean Sea, originating mainly from Algeria, Tunisia and Libya [14–17].

Northern Africa, the dominant sediment source, experienced drastic hydrological changes during the late Quaternary. These hydrological changes were driven by changes in the Northern Hemisphere summer insolation and the consequential migration of the Intertropical Convergence Zone (ITCZ) and the tropical rain belt over the African continent [18]. The ITCZ migrated northward during the Northern Hemisphere summer insolation maxima and caused considerably higher precipitation in North Africa. Large lakes and rivers existed and a dense vegetation cover characterised these so-called African Humid Periods (AHPs) [19–24]. The strongest AHPs are further characterised by the deposition of sapropel layers in the Eastern Mediterranean Sea. It should be noted that the AHPs lasted longer than the sapropels which represent the culmination of the AHPs [9,10]. Favourable environmental conditions in North Africa during the AHPs provided migration pathways for anatomically modern humans and supported their movement out of Africa and into the Mediterranean region [25,26]. The riverine sediment input into the Eastern Mediterranean Sea was much more pronounced during the AHPs than during the dry periods, when windblown dust was a more important sediment source [4,27–30]. Similar results were gained from the investigation of sediments from the northwest African continental margin, which is another sink for Saharan dust (e.g., [31,32]).

In this study, we use end-member modelling of high-resolution grain size, clay mineral and XRF core scanning data, which we extracted from sediment core M40/4_SL71 that was collected southwest of Crete (Fig 1). Our aim is to test whether end-member modelling of the individual datasets provides reasonable and coherent results for the reconstruction of sedimentation processes in the Eastern Mediterranean Sea during the last ~180 kyr.

## Materials and methods

The sediment core M40/4_SL71 investigated in this study was retrieved from the eastern Ionian Sea, south of the Hellenic Trench in the central part of the Eastern Mediterranean Sea (Fig 1). The core was recovered approximately 50 km SW of Crete (34°48.67’ N, 23°11.65’ E) at a water depth of 2788 m from the deep Mediterranean Ridge during cruise M40/4 of the German research vessel “Meteor” in 1998 [33]. Core retrieval was carried out in compliance with international law and regulations of the neighbouring countries as mediated by the “Control Station German Research Vessels” (formerly “Meteor Operations Control Office”) of the University of Hamburg. The core shows no evidence of sediment redistribution or hiatuses [29]. The core was previously used in various studies focusing on past alkenone and stable isotope sea surface temperatures and salinities [34], basin-wide water mass circulation and sediment provenance using Nd- and Sr-isotopes [5,6], and the dynamics of dust input into the Eastern Mediterranean Sea using clay minerals [29].

We analysed the upper 421 cm of the core covering a time span of ca. 182 kyr with an average resolution of ca. 220 years per sample. The age model of the core is taken from [29] and is based on the stable oxygen isotope record of surface-dwelling planktonic foraminifera (*G*. *ruber*, *G*. *bulloides*, *G*. *sacculifer* and *O*. *universa*), five ^14^C AMS dating results and the occurrence of tephra layer Y5 [35] (Table 1). For consistency, we adjusted the age model of the comparative core M51/3_SL110 from the southeast Levantine Sea (32°38.95`N, 34°06.22`E, 1437 m water depth) by correlating the δ^18^O record [10,36] with the LR04 stack [37] rather than with that of LC21 used in the original paper [10] (Table 1).

**Table 1:** Data used to construct the age model of the sediment core investigated in this study, M40/4_SL71 (reproduced from [29]), and to adjust the age model of the comparative core M51/3_SL110 [10].

Core	Depth (cm)	Age (cal ka BP)	Datum
**SL71**	0.00	0.000	sediment surface
**SL71**	2.50	0.2	δ^18^O record
**SL71**	4.75	1.935 ± 0.165	AMS ^14^C
**SL71**	13.25	6.865 ± 0.165	AMS ^14^C
**SL71**	15.00	7.8	δ^18^O record
**SL71**	20.25	10.825 ± 0.265	AMS ^14^C
**SL71**	35.00	17.9	δ^18^O record
**SL71**	36.25	18.790 ± 0.120	AMS ^14^C
**SL71**	50.25	25.540 ± 0.390	AMS ^14^C
**SL71**	84.00	39.280 ± 0.110	Top Y5 –Tephra
**SL71**	88.00	39.280 ± 0.110	Base Y5—Tephra
**SL71**	100.00	50.2	δ^18^O record
**SL71**	127.50	56.1	δ^18^O record
**SL71**	147.50	67.2	δ^18^O record
**SL71**	157.50	70.7	δ^18^O record
**SL71**	177.50	77.5	δ^18^O record
**SL71**	192.50	86.5	δ^18^O record
**SL71**	215.50	96.4	δ^18^O record
**SL71**	227.50	103.3	δ^18^O record
**SL71**	239.50	114.3	δ^18^O record
**SL71**	270.34	124.0	δ^18^O record
**SL71**	286.80	130.0	δ^18^O record
**SL71**	291.73	135.1	δ^18^O record
**SL71**	305.55	139.9	δ^18^O record
**SL71**	346.79	153.0	δ^18^O record
**SL71**	366.48	155.9	δ^18^O record
**SL71**	388.83	167.0	δ^18^O record
**SL71**	414.47	174.0	δ^18^O record
**SL71**	425.08	185.0	δ^18^O record
**SL71**	465.49	192.0	δ^18^O record
**SL110**	0.00	0.000	Sediment surface
**SL110**	34.50	6.43	AMS^14^C
**SL110**	64.50	11.53	AMS^14^C
**SL110**	113.50	17.8	δ^18^O record
**SL110**	150.50	24.4	AMS^14^C
**SL110**	234.50	33.9	AMS^14^C
**SL110**	464.50	66.0	δ^18^O record
**SL110**	529.50	86.9	δ^18^O record
**SL110**	579.00	108.9	δ^18^O record
**SL110**	598.00	115.0	Hiatus end
**SL110**	598.00	121.7	Hiatus start
**SL110**	603.00	124.0	Base S5
**SL110**	629.50	134.9	δ^18^O record

Age points are derived from AMS ^14^C dates, the age of the Y5-Tephra [35] and graphical correlation of the δ^18^O record of *G*. *ruber* (white) [10,36] with the LR04 stack [37].

Core M40/4_SL71 consists mainly of yellowish, brownish and greyish foraminifera-nannofossil ooze and includes five sapropel layers: S1 (10.2–8.0 ka), S3 (81.5–77.9 ka), S4a (98.5–97.1 ka), S4b (103.5–102.9 ka), S5 (126.6–121.3 ka), and S6 (174.2–164 ka). The bisection of sapropel S4 is also described in other sediment cores from the Eastern Mediterranean Sea (e.g., [9,38,39]).

Samples for grain size and clay mineral analyses were collected at a 0.5 cm spacing. The samples were treated with 10% acetic acid and 3% hydrogen peroxide to remove carbonate and organic carbon, respectively. Then, the clay fraction (<2 μm) was isolated from the coarser components by using settling tubes. Sieving through a 63 μm mesh separated the silt (2–63 μm) and sand fractions (>63 μm).

Clay mineral analyses followed standard procedures [40]. The percentages of smectite, illite, chlorite, kaolinite and palygorskite were calculated using empirical weighting factors [41,42]. The percentages of kaolinite and palygorskite had been published previously [29].

We performed detailed grain size analyses of the silt fraction using a Laser Particle Sizer (Analysette 22, Fritsch GmbH) with 31 silt fraction size classes. We measured each sample four times and calculated the mean grain size distribution for each sample. The standard deviation for each size class between the four measurements ranged between 0.035 μm and 0.053 μm, with a mean of 0.044 μm.

Major and trace elements were measured using an „ITRAX”(Cox Analytical Systems) XRF core scanner equipped with a Cr-tube running at a tube voltage of 30 kV and a tube current of 30 mA. The exposure time was 15 s per measurement, and the scanning interval was 0.2 cm. The core scanning data must be interpreted carefully, because no data for calibrating and correcting for the water content are available. Of all detectable elements, Si, K, Ti, Fe, Mn, Rb, Zr and Ba were considered for the modelling. Si, K, Ti, Fe, Mn, Rb and Zr describe the basic chemical composition of the terrigenous sediment fraction. In contrast, Ba can be either detritic or biogenic: during sapropel deposition, the biogenic proportion is greater than the detritic proportion.

We calculated the ratio of the intensity counts per second of each element to the total number of counts of the group of selected elements. A z-transformation for standardisation was then applied to the dataset.

The clay mineralogy, grain size and XRF scanning datasets were fed separately into the open source *R*-based end member composition algorithm RECA [43] to identify similarities in the variation patterns of the individual variables within each dataset. Based on the similarities of the variation patterns, the algorithm groups the individual variables into a predefined number of subpopulations (end members). The determination of the number of end members is an iterative process in which the dataset is simplified to a minimum number of end members that can explain a large portion of the variance in the dataset. The number of end members is chosen based on geological plausibility and the coefficient of determination (*r*^2^ mean) between the original data and the dimensionally reduced modelled matrices ([35]; and references therein). If an increase in the number of end members did not significantly improve the *r*^2^ mean, the lower number of end members was selected [43,44]. RECA was run in R, version 3.0.0 [45].

The composition of each end member is defined by the eigenvalues of the variables. High eigenvalues mean that the variable is strongly represented by the end member. The end member loadings through time indicate the proportion of the overall variance in the dataset that the end members describe. At each timestep, the loadings of all end members in the model sum up to 1.

To compare the results of the end-member modelling to a widely used and classical approach, we additionally applied an R-Mode principal component analysis (PCA) using a combination of the three datasets. To reduce the size of the dataset, only seven grain size classes, represented in the three modelled end members, were used for the PCA. Similar to the end-member modelling, the PCA is searching for principal components (PCs) that are linear combinations of the original variables and explain the most variance in the original dataset. The calculations are based on the correlation matrix of the original datasets and was performed using the software package Past, version 2.15 [46].

The raw data used in this study are stored in the Pangaea data base at https://doi.pangaea.de/10.1594/PANGAEA.879595.

## Results

**Fig 2:**
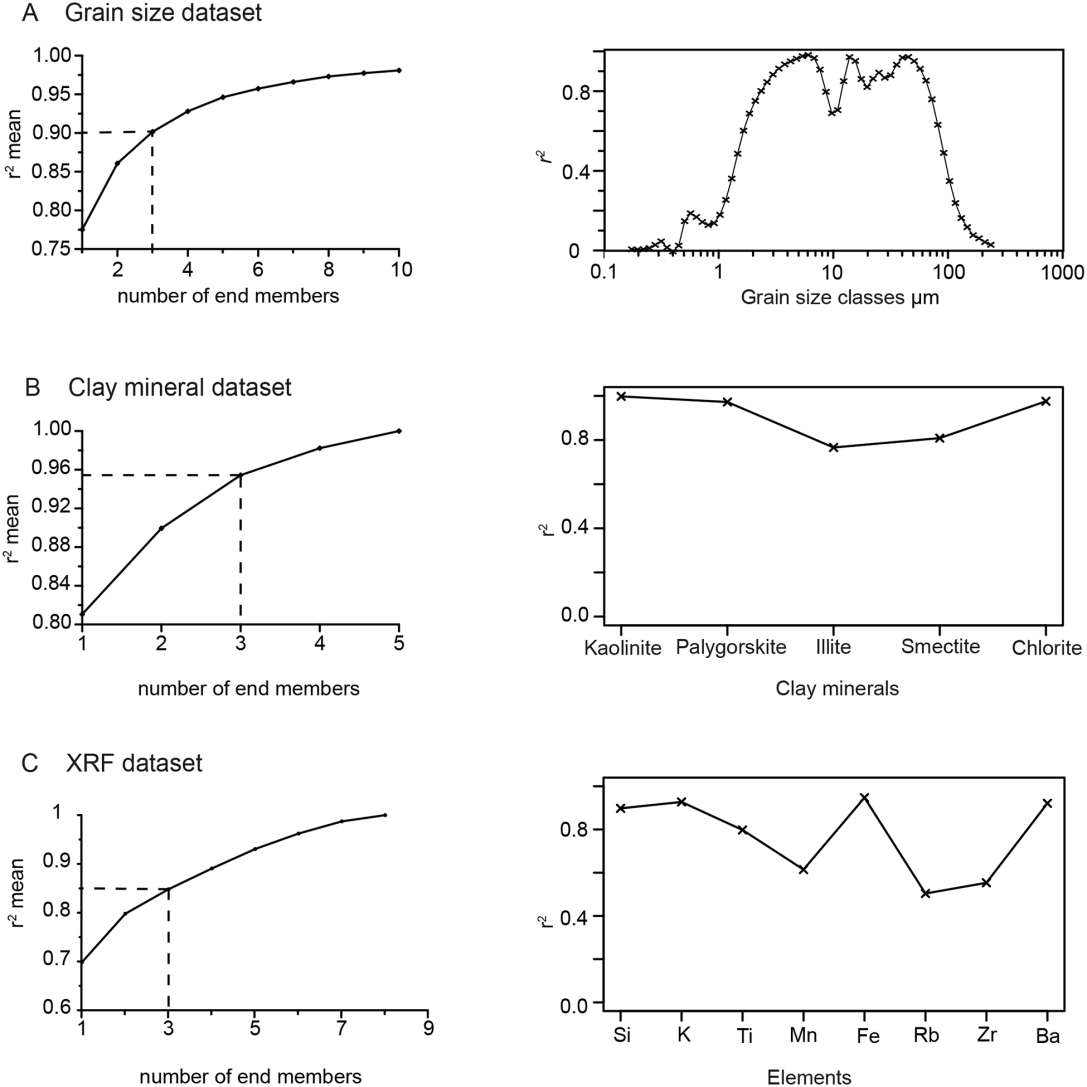
Results of the end-member modelling of the three datasets from sediment core M40/4_SL71. (A) grain size dataset, (B) clay mineral dataset, (C) XRF dataset. Left: *r*^2^ mean = mean coefficients of determination versus the number of end members in each end-member model. Right: *r*^2^ = coefficient of determination for each variable used in the end-member modelling.

Based on the coefficient of determination (*r*^2^ mean), a three-end-member model was chosen for each dataset. The models explain 90.0% of the variance in the grain size dataset, 95.4% of the variance in the clay mineral dataset and 84.5% of the variance in the XRF dataset (Fig 2).

**Fig 3:**
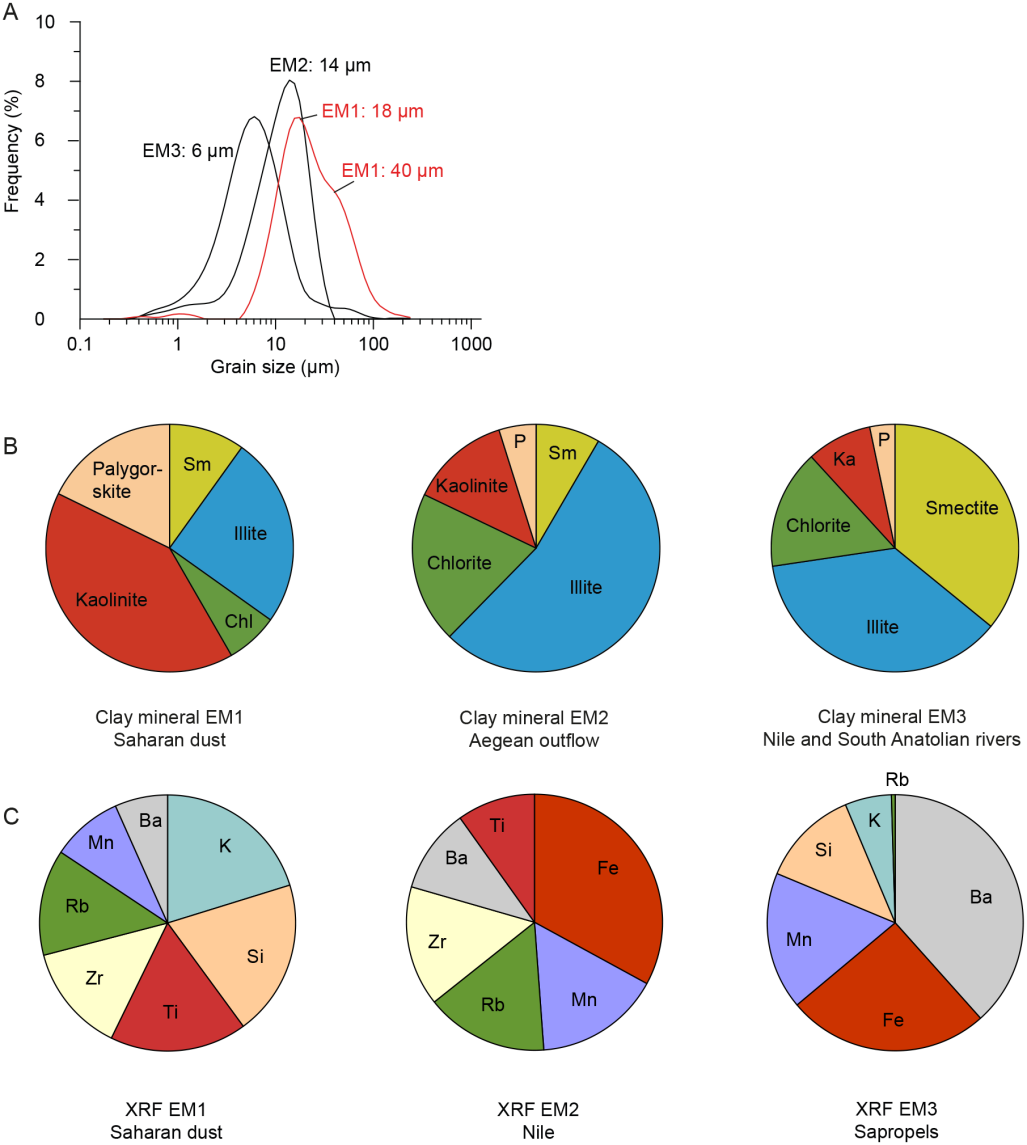
Composition and grain sizes of the end members in core M40/4_SL71. A: grain size end members, B: clay mineral end members, C: XRF end members.

The three end members of the silt grain size data (grain size EM) differ only slightly with respect to their modal grain sizes (Fig 3A). Grain size EM1 has two modes, a dominant mode of approximately 18 μm and a subordinate mode of approximately 40 μm. Grain size EM2 and EM3 are unimodal, with modal grain sizes of 14 μm and 6 μm, respectively.

The compositions of the three clay mineral end members (clay mineral EM) are illustrated in Fig 3B. Clay mineral EM1 is dominated by kaolinite (41%), palygorskite (18%) and illite (25%). Smectite (10%) and chlorite (7%) are only minor components. Clay mineral EM2 is dominated by illite (54%). Additionally, this end member has a high contribution of chlorite (20%). Kaolinite (13%), smectite (8%) and palygorskite (5%) contribute minor proportions. Clay mineral EM3 is characterised by the highest proportion of smectite (36%) of all end members and a similarly high contribution of illite (37%). Chlorite, kaolinite and palygorskite account for only 16%, 9% and 3% of EM3, respectively.

The compositions of the geochemical bulk sediment end members (XRF EM) are illustrated in Fig 3C. XRF EM1 is dominated by K (20%), Si (19%) and Ti (17%) with moderate contributions of Zr (14%) and Rb (13%). Mn (9%) and Ba (6%) only contribute minor proportions. XRF EM2 has the highest proportion of Fe (33%), a moderate contribution of Mn (16%), Rb (15%) and Zr (15%) and a minor contribution of Ba (11%) and Ti (9%). XRF EM3 is primarily characterised by Ba (38%), Fe (25%) and Mn (17%) with moderate contributions of Si (12%), K (6%) and Rb (0.5%).

## Discussion

### Assignment of end members

#### Grain size patterns and assignment of grain size end members

The Saharan dust shows mean modal and median grain sizes of 5–30 μm [17]. A grain size range of 1–100 μm for Mediterranean aerosols was previously reported [47], with modal sizes of 10–30 μm. A grain size mode of 40 μm was measured in sediments of the Nile margin, and was related to Saharan dust [4]. Additionally, typical median grain sizes of approximately 16 μm [15], 4–16 μm [48] and 8–30 μm [49] were reported for modern Saharan dust samples collected on the island of Crete and in the surrounding sea adjacent to the study site M40/4_SL71. Some grain size studies also used end-member modelling. Thus, in late Quaternary sediments off the coast of Israel, an end member with a mode of 40 μm was interpreted to describe the Saharan dust, and two end members with modes of 4 μm and 10 μm were interpreted to describe the fluvial/Nile sediments [7]. In the Nile deep-sea fan, a bimodal end member with modes of 15 μm and 30 μm was interpreted to represent the Saharan dust, whereas two end members with 2 μm and 3–4 μm were interpreted to represent the Nile discharge [8]. Additionally, a Saharan dust end member of 40 μm and fluvial end member of 10 μm were reported for samples collected off the northwest African coast [31].

**Fig 4:**
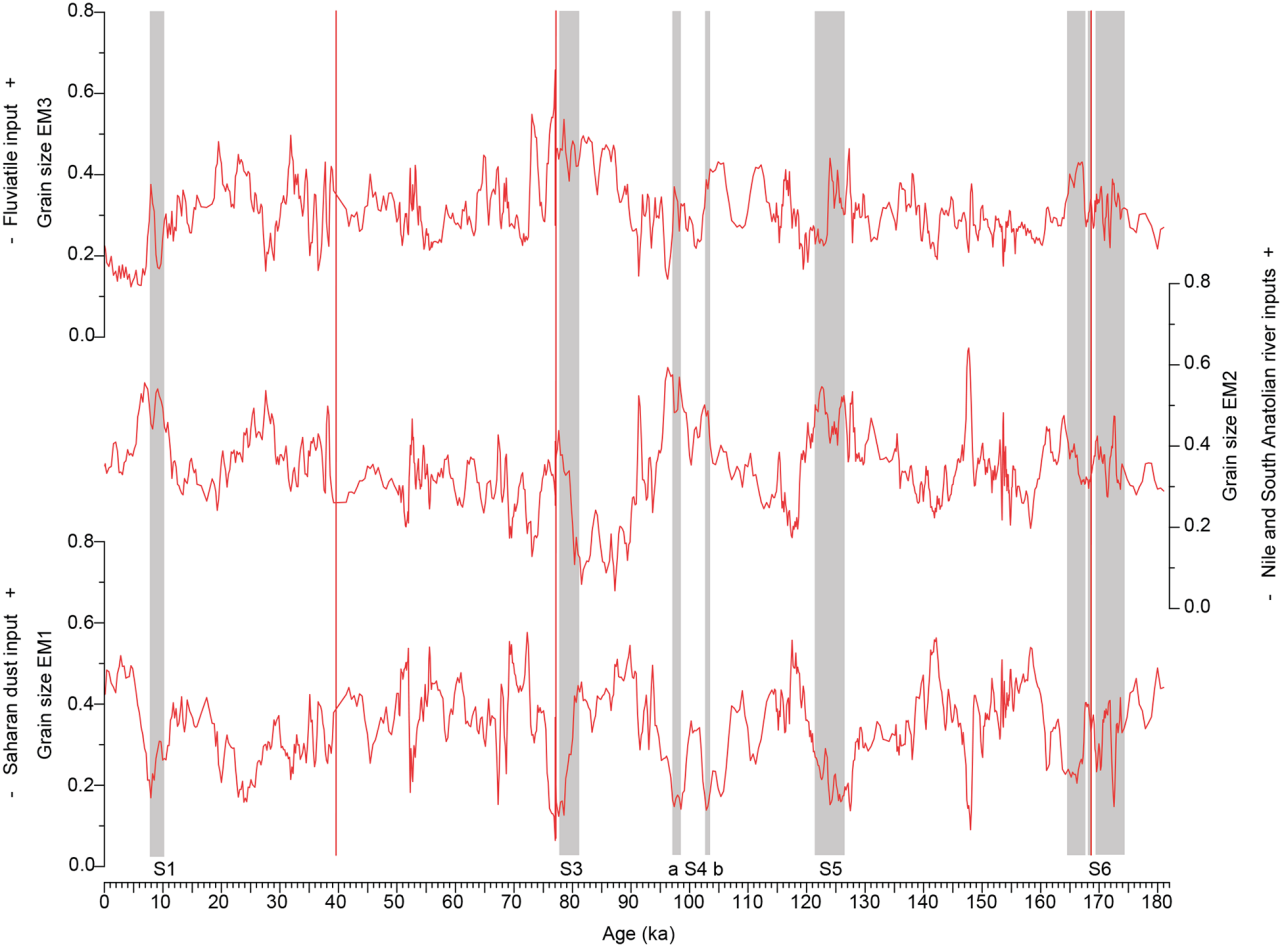
Variation patterns of the grain size end-member loadings in core M40/4_SL71 through time. Grey shaded areas mark the sapropels, and the ash layers are indicated by the red lines.

According to the aforementioned studies, our modal grain sizes of EM1 are typical for Saharan dust blown into the Eastern Mediterranean Sea (Fig 3A). The results from the grain size EM1 show especially high loadings during the dry phases and low loadings during the humid phases (Fig 4). The bimodal character of this end member may hint to the existence of multiple source areas for dust from the Sahara, characterised by different grain size distributions. It may also indicate variations in the intensity of dust delivery due to seasonally variable wind activity [17,50], but this seasonal variation cannot be resolved with a 220 years/sample resolution. The alternative explanation that the bimodal character of the grain size EM1 is a combination of a coarser aeolian and a finer fluvial sediment component transported to the core site at the same time is unlikely. It can be expected that the variation patterns of fluvial and aeolian input are negatively correlated to each other because they are driven by different climatological conditions; thus they would not be assigned to the same end member.

The modes of the grain size EM2 and EM3 are 14 μm and 6 μm, respectively, and these grain sizes can be expected for fluvial sediments in a distal position in the basin [51]. The grain size EM2 generally increases during humid phases with sapropel formation (Fig 4). This finding corroborates our interpretation of grain size EM2 as fluvial input. The variation pattern in the loadings of grain size EM3 is less pronounced. The loadings tend to increase during the humid phases, but no clear trend can be identified. However, the modal size is approximately the fluvial end member in the eastern Levantine Sea [7]. Thus, our grain size EM3 likely represents fluvial sediment influx as well.

#### Provenance of the clay minerals and assignment of the clay mineral end members

End-member modelling of the clay mineral data groups together illite, chlorite, smectite, kaolinite and palygorskite in different proportions (Fig 3B), indicating distinct sediment sources. The provenance of the clay minerals deposited in the Eastern Mediterranean Sea is relatively well known from studies that have mapped their distribution patterns in seafloor surface sediments [12,52–57] and in sediment cores [9,10,40,54,57–61] and by analysing aeolian dust [15,62–64].

According to these studies, southern European rivers that discharge into the western Aegean Sea are the primary sources of illite, while the Anatolian rivers are minor contributors [40]. Furthermore, the Po delivers a large amount of illite to the Adriatic Sea [55]. Ocean currents may transport the illite from these regions into the Ionian Sea. Illite is also a component of the aeolian dust blown out of North Africa. Kaolinite derives dominantly from North Africa and is transported toward the Eastern Mediterranean Sea either by wind from the Sahara or by the Nile from the Egyptian wadi areas [52,57]. Palygorskite is a typical wind-blown clay mineral in Eastern Mediterranean Sea sediments and an excellent tracer for Saharan dust derived from specific areas in Morocco, Algeria, Tunisia, Libya and Egypt [54,64]. Smectite is mainly sourced from the Nile and is transported from the southeastern Levantine Sea to the region east of Crete by the surface currents along the coasts of Israel, Lebanon, Syria and Turkey [52,54]. Minor smectite sources are the outflow of the Black Sea and the Anatolian rivers that discharge into the eastern Aegean Sea and the northern Levantine Sea. Smectite may also be derived from the Sicilian coast and the Adriatic Sea. In contrast to the other clay minerals, no distinct source is evident for chlorite. This clay mineral is ubiquitous in minor amounts in the seafloor surface sediments of the Eastern Mediterranean Sea [40,52,57]. Except for palygorskite all clay minerals are present in each potential source area. This findings explains why these minerals are present in all clay mineral end member compositions but in different proportions (Fig 3B).

**Fig 5:**
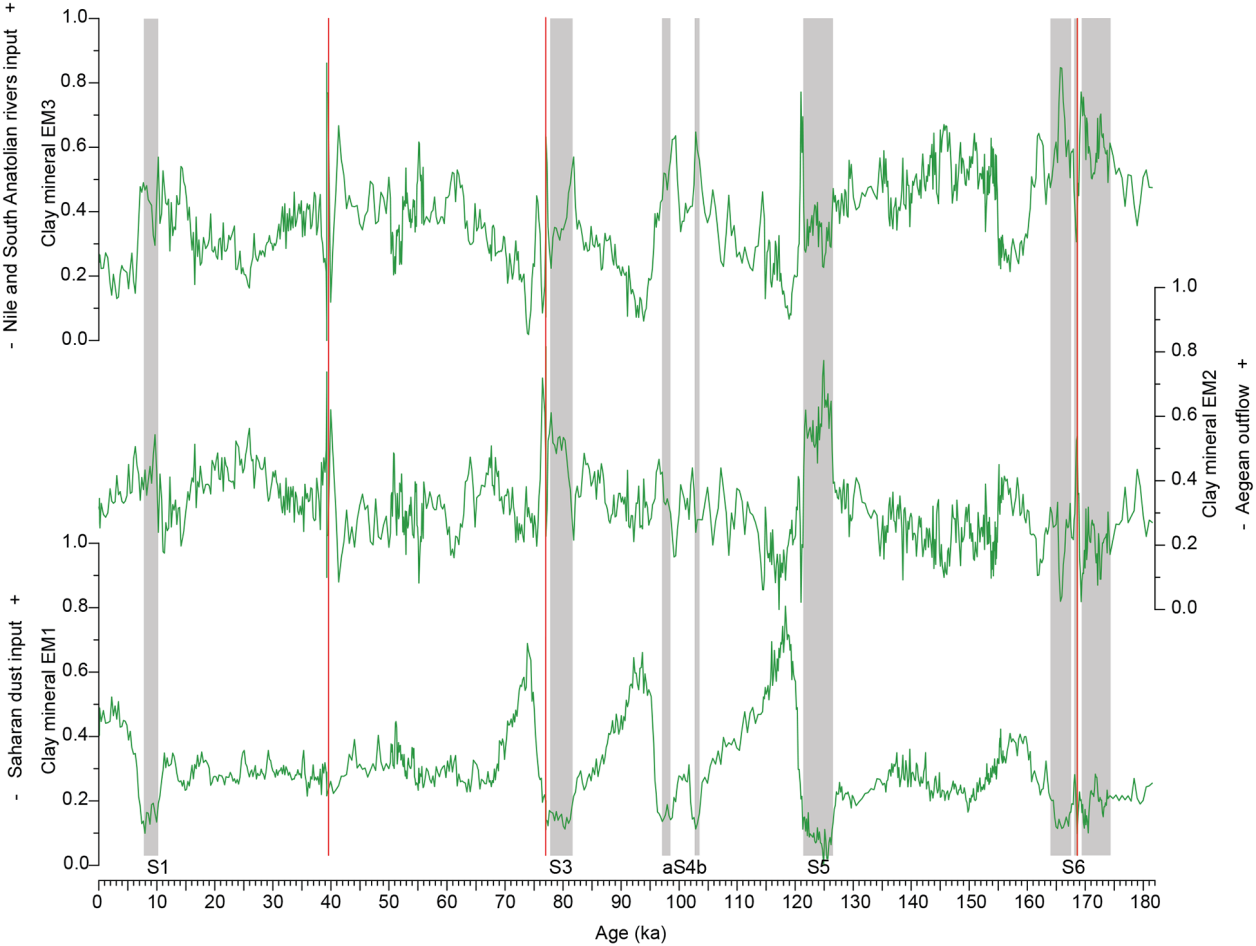
Variation patterns of the clay mineral end-member loadings in core M40/4_SL71 through time. Grey shaded areas mark the sapropels, and the ash layers are indicated by the red lines.

Clay mineral EM1 is characterised by kaolinite, palygorskite and illite. It clearly represents the influx of North African dust because this is the only source for palygorskite. Additionally kaolinite and illite are common components of Saharan dust [17,62,63]. The temporal distribution pattern of clay mineral EM1 in M40/4_SL71 (Fig 5) is nearly identical to the percentages of kaolinite and palygorskite and to the kaolinite/chlorite ratios used to quantify the Saharan dust input into the Eastern Mediterranean Sea [29].

The clay mineral EM2 has a similar signature as the sediments discharged by the NW Aegean rivers, which are characterised by approximately 60% illite, 20% smectite, 10% chlorite and 10% kaolinite [12]. A similar assemblage is found in the Kithira Strait between Peloponnese and Crete, which is the outflow path of the Aegean surface water [52]. We therefore assume that clay mineral EM2 represents the sediment input from the western Aegean Sea by the surface currents describing a counter-clockwise gyre in the Aegean Sea (Fig 1). The clay mineral EM2 also contains minor proportions of palygorskite and kaolinite, which can be explained by minor amounts of Saharan dust blown to the Aegean Sea [9] and then transported southward into the Ionian Sea by the surface currents.

The composition of clay mineral EM3 is dominated by smectite and illite, which contribute 36% and 37%, to this end member, respectively (Fig 3B). The clay fraction of the seafloor surface sediments east of Crete contains approximately 40–50% smectite and 30–40% illite [52], which is similar to the composition of the clay mineral EM3. According to earlier clay mineral analyses of surface sediments in the Eastern Mediterranean Sea, there are two possible source areas for EM3: (i) the Nile, which is the main supplier of smectite to the Eastern Mediterranean Sea, and (ii) the South Anatolian rivers, whose clay mineral compositions show similar smectite and illite concentrations [52] as found in clay mineral EM3. The Nile clay mineral assemblage in the southeastern Levantine Sea consists of approximately 70% smectite, 20% kaolinite, <10% illite and traces of chlorite [57]. The present-day Nile suspension load follows the counter-clockwise surface currents along the coast of the Near East and Turkey to the region east of Crete (Fig 1). This finding is corroborated by the ^143^Nd/^144^Nd and ^87^Sr/^86^Sr fingerprints of the lithogenic seafloor sediments, which allow the tracking of Nile particulate matter to the NE-trending eastern flank of the Mediterranean Ridge. This is due to the higher _Ɛ_Nd^(0)^ values (-6 to -2) and lower ^87^Sr/^86^Sr ratio (0.707–0.712) of the Nile suspension load compared to the other sediment sources in the Eastern Mediterranean Sea [5]. Anatolian rivers discharging into the northern Levantine Sea dilute the Nile suspension and thus decrease its typically high smectite/illite ratio. Unfortunately, the isotopic signature of the erosion products of the Aegean basalts in the Anatolian region is similar to the Nile signature, which prohibits a distinction between the two source areas based on isotope data [5]. Currently, the Nile suspension load does not reach the region around Crete nor the M40/4_SL71 study site [5,6,52,54,65].

Within the sapropels, the _Ɛ_Nd^(0)^ values increase in the M40/4_SL71 core from approximately -11.5 to -7, and the ^87^Sr/^86^Sr ratios decrease from ca. 0.717 to 0.712 [6]. This pattern indicates an enhanced influx of Nile and/or South Anatolian sediment. A sediment influx from the west, e.g., from the Adriatic Sea or from the Sicilian coast, can be excluded due to its markedly different isotopic signature. Thus, we interpret that clay mineral EM3 indicates a Nile and/or South Anatolian sediment input.

#### Chemical composition and assignment of the bulk sediment end members

Numerous studies on the composition of the sediments from the Eastern Mediterranean region showed that various source areas have differing geochemical fingerprints and that geochemical data therefore can be used to reconstruct the sediment provenance [4,5,27,36,66–73]. To summarise, sediments coming from the Adriatic Sea have high K and Mg contents, while sediments coming from the Aegean Sea are characterised by Mg, Cr, N and minor K contents. Sediments from the Anatolian region have high contents of Ti and lower contents of Mg and K. The Nile discharges sediments rich in Fe and Al. Northern African dust typically has high Si, Ti, K and Zr contents. In contrast, the sapropel layers are characterised by enhanced concentrations of S, Fe and trace elements such as Ba, Co, Cr, Ni, V, Mo, Zn and Br.

**Fig 6:**
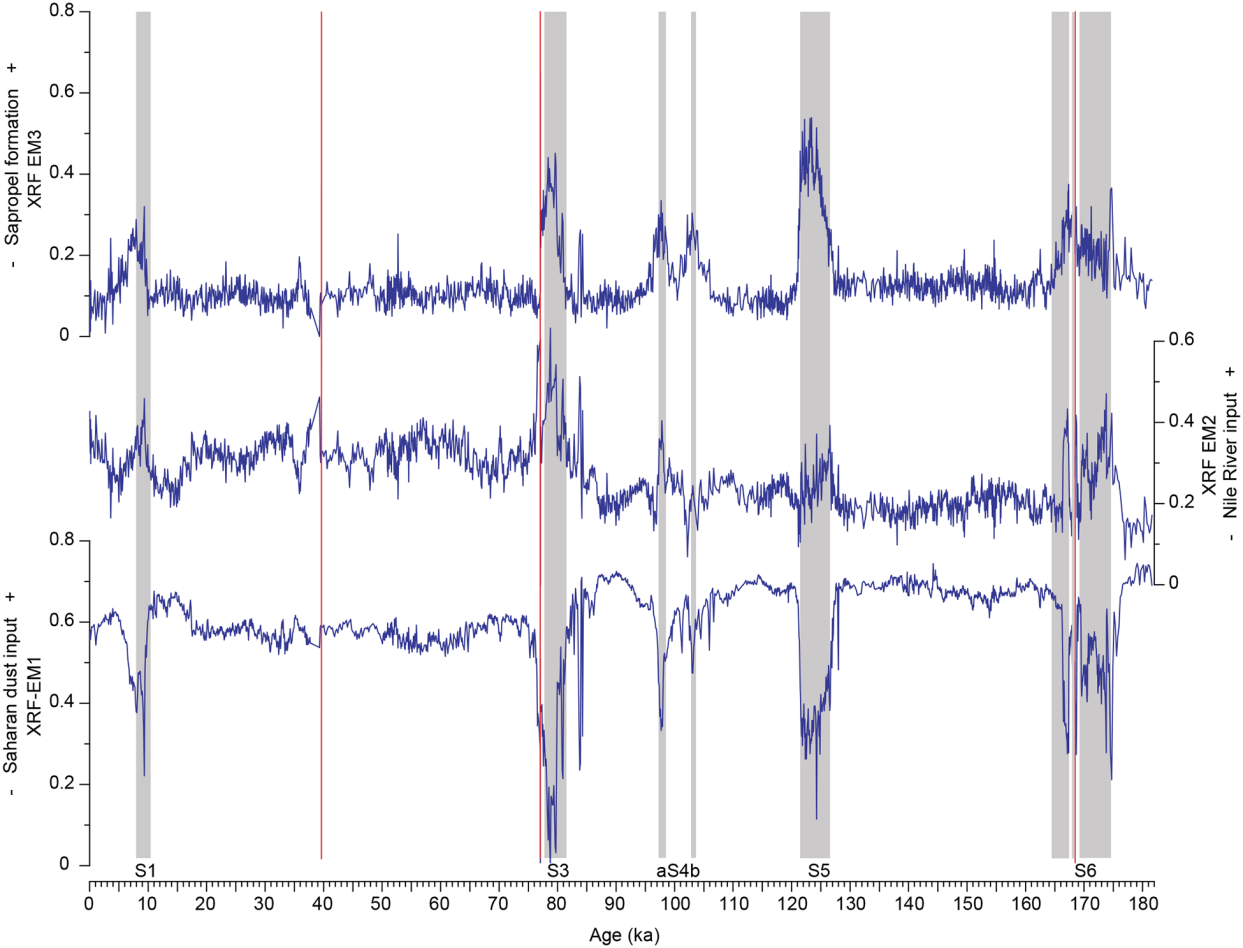
Variation patterns of the XRF end-member loadings in core M40/4_SL71 through time. Grey shaded areas mark the sapropels, and the ash layers are indicated by the red lines.

XRF EM1 is characterised by a predominance of K, Si and Ti (Fig 3C) and therefore represents the input of Saharan dust [27]. Moderate contributors to XRF EM1 are Zr and Rb. These two elements are also associated with Saharan dust [27,70,72]. XRF EM2 is dominated by Fe with minor contributions of Mn, Rb and Zr. XRF EM2 most likely represents Nile-derived sediments [4]. XRF EM3 is dominated by Ba and Fe. Ba is clearly associated with the deposition of anoxic, organic-rich sapropel layers [36,70] and is an indicator of bioproductivity and organic matter fluxes [36]. Fe could (i) be associated with the fixation of redox-sensitive elements due to prevailing anoxic conditions or (ii) represent an increased riverine sediment input from the Nile. The strict confinement of high XRF EM3 loadings to the sapropel layers (Fig 6) rather favours the former interpretation.

**Fig 7:**
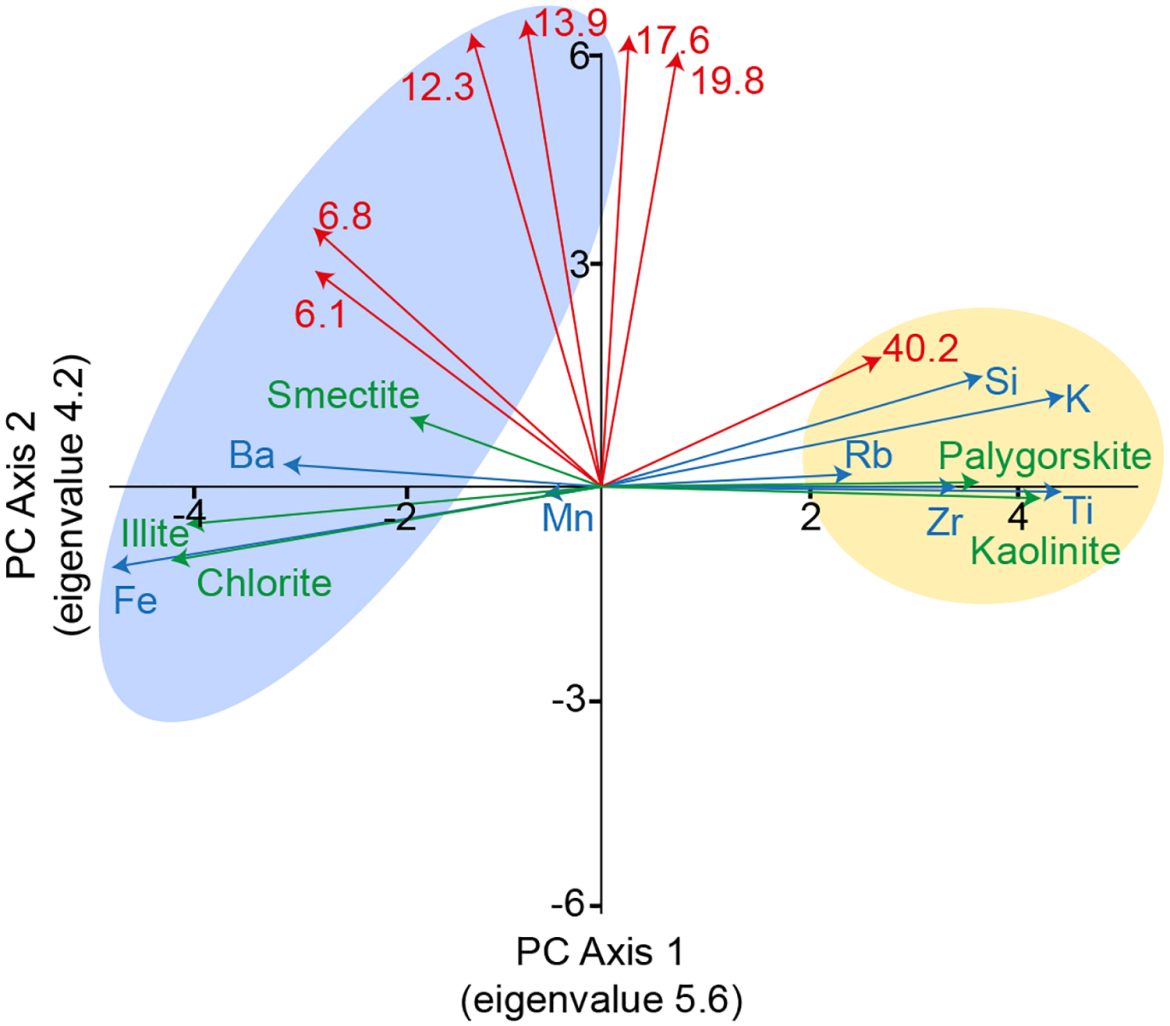
Results of the PCA of the combined dataset. The results for the grain size data are presented in red (numbers indicate grain size classes), the results for the clay mineral data are presented in green, and the results for the XRF data are presented in blue. Sediment parameters indicating Saharan dust are shaded in yellow, and the sediment parameters indicating fluviatile sediment influx are shaded in light blue. The eigenvalues of the PC axes are included in brackets.

The PCA results are shown in Fig 7. The first two axes explain approximately 49% of the variance in the dataset (axis 1: 28.1%, axis 2: 21.3%). The sediment parameters indicating North African dust (Si, K, Ti, Rb and Zr; 40 μm grain size; kaolinite and palygorskite) are grouped together and are clearly separated from the other data. The sediment parameters connected to the fluviatile sediment input (grain sizes: 6.8 μm, 6.1 μm, 12.3 μm and 13.9 μm; Ba, Fe, illite and smectite) show a broader distribution but are clearly separated from the dust parameters. However, a separation of the different fluvial signals (Nile and Anatolian region, Aegean outflow and sapropel formation) is not possible based on the PCA results.

### Temporal variations of the dust input into the Eastern Mediterranean Sea

**Fig 8:**
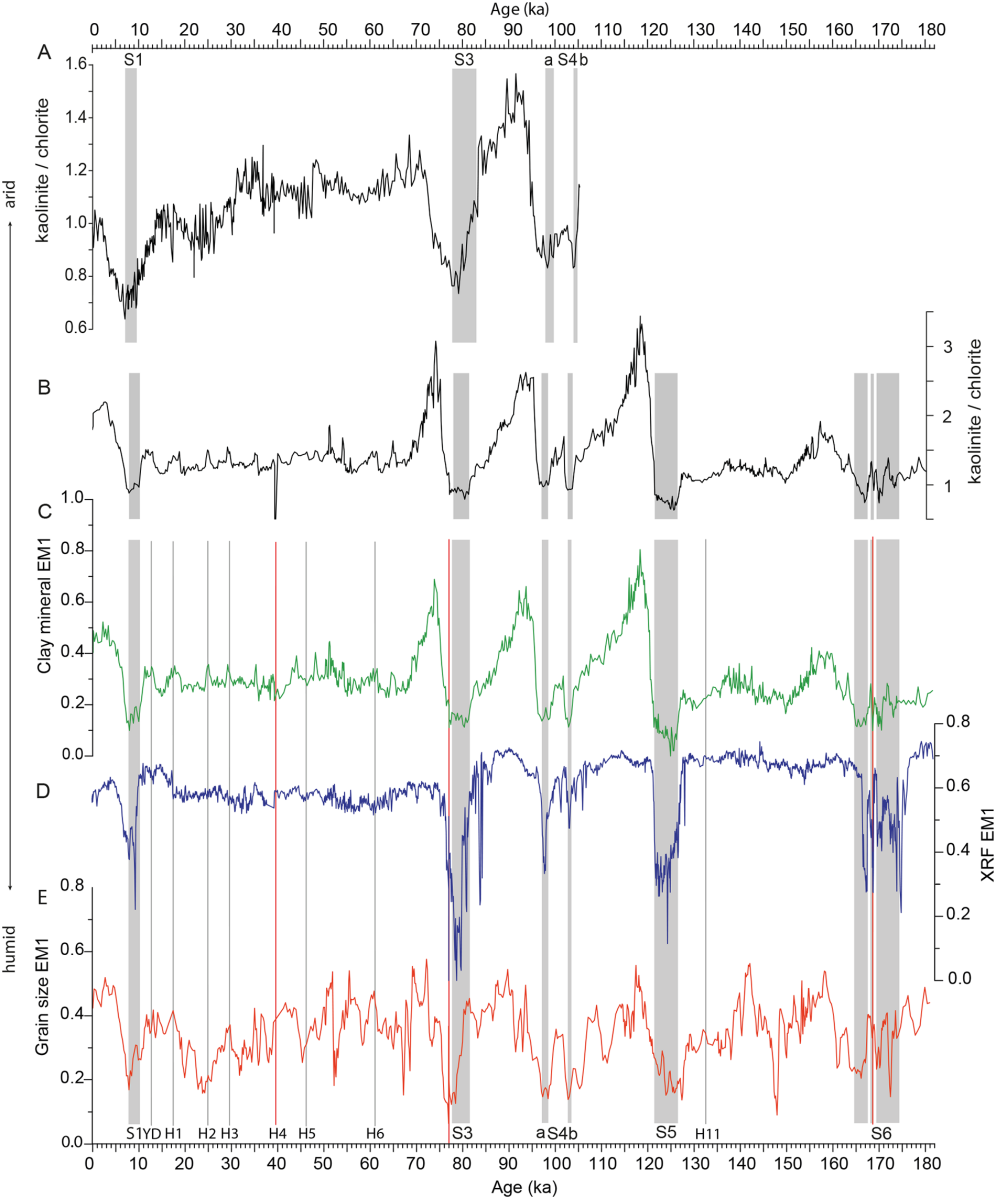
Proxies for the Saharan dust influx. A: kaolinite/chlorite ratio in sediment core M51/3_SL143 from the central Aegean Sea [9]; B: kaolinite/chlorite ratio in sediment core M40/4_SL71 [29]; C-E: Saharan dust end members in core M40/4_SL71; C: clay mineral EM1; D: XRF EM1; E: grain size EM1. Grey shaded areas indicate the sapropels, the red lines indicate the ash layers, and the grey lines mark the Northern Hemisphere cooling events. YD: Younger Dryas; H1-6, H11: Heinrich events.

An essential limitation of the interpretation of the contribution of individual end members is the problem of dilution because the loadings of the three end members of each dataset sum to 1.0. Therefore, a change in the loading of one end member causes changes in the loadings of the others. However, in our case the problem is mitigated. Our clay mineral dust end member is almost identical to the kaolinite/chlorite curve of the core [29]. In the study by [29] it was proven that the changes in the kaolinite/chlorite ratio result from variations in the kaolinite content and thus variations in the Saharan dust influx. Therefore, our clay mineral dust end member is definitely controlled by the influx of Saharan dust. The same is true for the other dust end members that correlate with the clay mineral dust end member (Fig 8). The correlation of our fluvial end members with independent data sets from the literature (see below) further indicates that the changes in the loading patterns of the end members are not caused by dilution.

Each dataset yielded an end member representing the input of Saharan dust into the Eastern Mediterranean Sea: clay mineral EM1, grain size EM1 and XRF EM1 (Fig 8). All dust end members show minimum loadings during the AHPs (Fig 8). During these episodes, the insolation-driven northward shift of the tropical rain belt caused an increase in the precipitation over northern Africa. Numerous and large lakes and rivers were established [20,21], and a dense vegetation cover formed [19,23,74], hampering the deflation of dust out of the Sahara.

During arid phases, the loading patterns differ between the individual dust end members. This finding can be explained by the different dust-controlling factors responsible for each dataset. Clay mineral EM1 represents fine-grained dust <2 μm in size. The typical feature of this end member is a drastic and sudden increase in the loadings to approximately 0.7 to 0.8 at the terminations of the humid phases, immediately followed by a gradual decrease to a base level with loadings of 0.2 to 0.3 (Fig 8). This pattern is almost identical to that of the kaolinite/chlorite ratio [29], which reflects the Saharan dust influx. Therefore, the same line of interpretation can be followed. During the humid periods, fine-grained erosion products accumulated in the North African lakes and soils. At the transition from the humid to arid phases, the lakes and soils desiccated and created the kaolinite- and palygorskite-rich, fine-grained sediments available for mobilisation by wind. Therefore, the flux of clay-sized aeolian dust increased abruptly and drastically for a relatively short time. The clay input decreased again when the sediment sources gradually exhausted and remained low through the end of the arid period.

The kaolinite/chlorite record in sediment core M51/3_SL143 from the Aegean Sea exhibits a similar pattern of clay-sized Saharan dust influx [9]. Additionally, in this core, the dust influx shows distinct minima during the AHPs, followed by a strong and rapid increase (Fig 8). However, the dust influx in core M51/3_SL143 is on a lower level compared to the dust influx in core M40/4_SL71. This finding can be explained by the more distal setting of M51/3_SL143.

The loadings of clay mineral EM1 decrease to almost 0 briefly within sapropel S5. The other dust end members show a strong decrease in their loadings but do not decrease to 0 during this period. This finding suggests that the dust input from North Africa during this humid period is greatly reduced but did not completely stop. The humid period associated with S5 is the most intense humid phase documented in this core [29]. The collapse in the loadings of clay mineral EM1 suggests that no desiccated lakes or soils were available as a source for fine-grained, kaolinite- and palygorskite-bearing dust due to high humidity.

Grain size EM1 represents the silt-sized dust of the sediments. In contrast to clay mineral EM1, grain size EM1 shows greater short-term fluctuations at a higher base level (approximately 0.3) during the arid phases. XRF EM1 represents a dust signal based on the bulk sediment chemistry. XRF EM1 remains at a high base level (between 0.5 and 0.7) and only decreases during the humid periods. High loadings of grain size EM1 correlate with the Heinrich events [75,76], which are characterised by stronger winds and “mega droughts” in northern Africa and therefore a higher dust export from the continent [77,78] (Fig 8). The Heinrich events are also evident in clay mineral EM1, although in a lesser form, because the main sediment source for the clay-sized dust was mostly exhausted during these times. Unlike clay mineral EM1, which is controlled by the availability of fine-grained material, we assume that wind strength is the controlling factor for grain size EM1 and XRF EM1 because their sediment sources were never exhausted.

The grain size dust end member does not mirror the position of sapropel S3 as precisely as the other dust end members possibly because different source areas and transport mechanisms are responsible for the individual end members.

### Temporal variations of the fluvial input into the Eastern Mediterranean Sea

**Fig 9:**
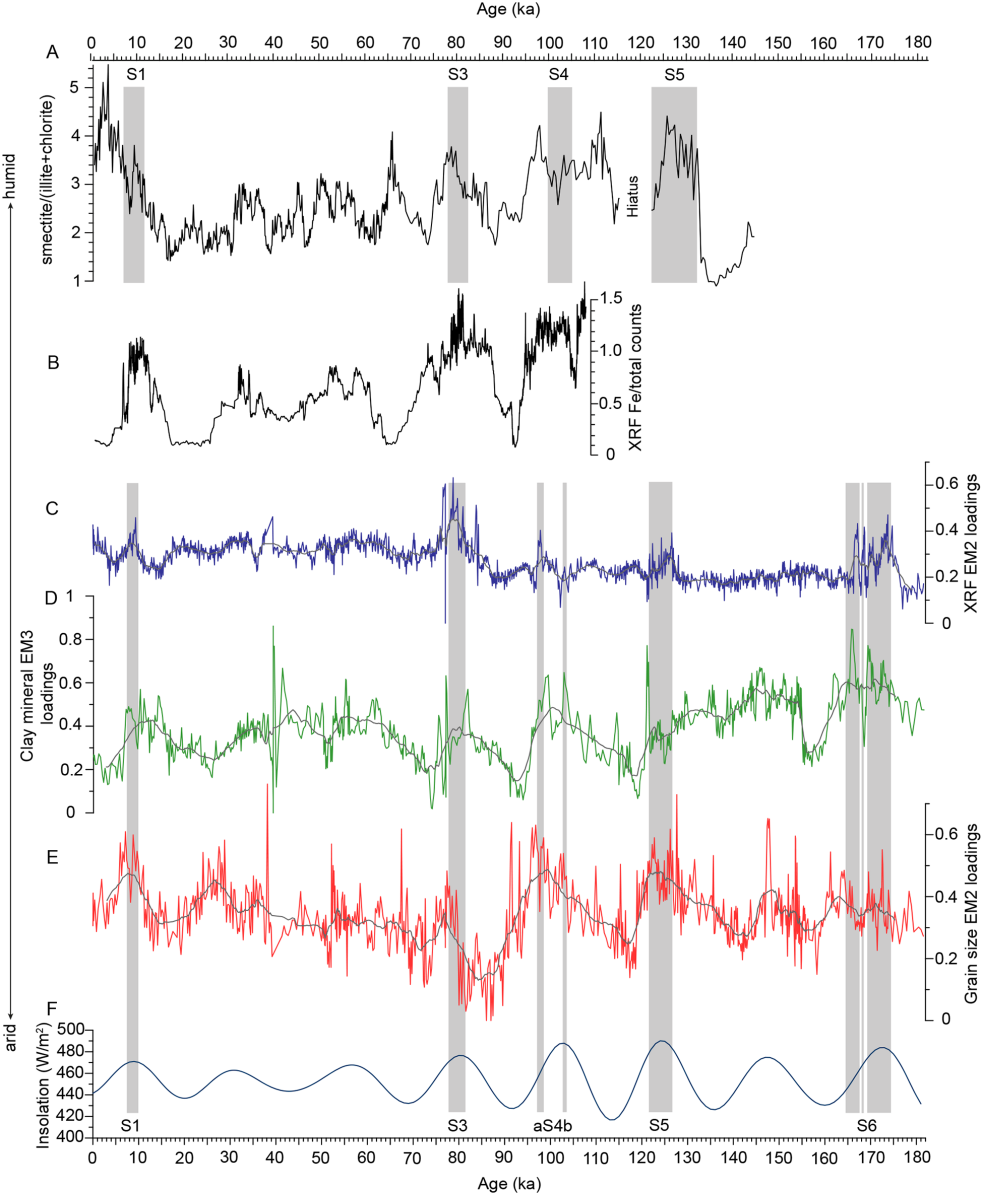
Compilation of proxies for the fluvial discharge into the Eastern Mediterranean Sea. A: Nile discharge based on smectite/(illite + chlorite) in sediment core M51/3_SL110 from the southeast Levantine Sea [10]; B: Nile discharge in sediment core MS27PT based on Fe/total counts data from the Nile [4,80]; C: XRF EM2 from M40/4_SL71, D: clay mineral EM3 from M40/4_SL71; E: grain size EM2 from M40/4_SL71, F: summer insolation curve (21 June, 30° N) [79]; Black line in C—E: 25-point running average. Grey shaded areas indicate the sapropels.

Clay mineral EM3, grain size EM2 and XRF EM2 are interpreted as representing fluvial input from the Nile and/or South Anatolian rivers. Since these end members show very similar variation patterns, we assume that they are influenced by the same environmental factors. The temporal variations in the loadings of clay mineral EM3 and grain size EM2 correlate well with the Northern Hemisphere summer insolation [79] (Fig 9). High loadings of these end members are associated with the strong insolation maxima that led to the AHPs, with a high fluvial input which triggered sapropel formation in the Eastern Mediterranean Sea. High loadings of clay mineral EM3 and grain size EM2 also occur during the weaker insolation maxima at 31 ka, 57 ka and 147 ka, which did not result in sapropel formation. An enhanced Nile runoff during insolation maxima is also recorded by the Fe content in the Nile margin sediments [4,80], with distinct maxima during times of sapropel S1, S3 and S4 formation, and weaker maxima at 31 ka and 57 ka.

The interpretation of an enhanced Nile runoff and higher sediment input from the South Anatolian region during the weak insolation maxima at 31 ka, 57 ka and 147 ka is based on the chemical composition of sediments and is also documented by [27]. Low loadings are associated with the insolation minima and the resulting dry phases in northern Africa. Thus, clay mineral EM3 and grain size EM2 seem to be influenced by fluvial input and therefore may indicate changes in humidity in the Mediterranean region and northern Africa. XRF EM2 shows a less distinct correlation with the Northern Hemisphere insolation but has high loadings during the humid phases. The large Fe contribution to the composition of this end member hints to the Nile as the main sediment source [4] rather than the South Anatolian rivers (Fig 9).

As mentioned before, the suspension load of the Nile currently does not reach the core site [6,52,65]. We assume that during times of increased Nile runoff, the sediment suspension load was transported farther and did reach the core site. However, enhanced humidity and therefore enhanced fluvial sediment input from South Anatolian rivers is known to be in phase with the monsoon-influenced climate system of Africa [81]. Consequently, the fluvial suspension load of the Nile is likely diluted by the Anatolian rivers discharging into the northern Levantine Sea.

It is remarkable that all major changes in the Nile runoff reported in the proximal settings [4,10] are also noticeable in the fluvial end members of the much more distal sediment core M40/4_SL71 (Fig 9). This finding suggests that our fluvial end members document changes in fluvial influx rather than changes in the strength of ocean currents responsible for the sediment transport to our core site.

## Conclusions

Datasets composed of sedimentological and geochemical variables on the sediment composition of core M40/4_SL71 were subjected to end-member modelling in order to reduce them to a few end members. We calculated three end members for each of the clay mineral, the grain size and the bulk sediment XRF datasets. This calculation facilitated the identification of similarities between the fluctuation patterns of the variables and enabled a more holistic and robust interpretation of the processes associated with the late Quaternary alternation between arid and humid phases in North Africa and the eastern Mediterranean region.

We were able to assign all end members to different source areas and/or transport mechanisms. Three end members represent the dust export from the Sahara (grain size EM1, clay mineral EM1 and XRF EM1). Five end members pertain to riverine sediment discharge into the Eastern Mediterranean Sea (grain size EM2 and EM3, clay mineral EM 2 and EM3 and XRF EM2). XRF EM3 is associated with the fixation of redox-sensitive elements in the sapropel layers reflecting bottom-water anoxia and possibly enhanced organic matter fluxes during sapropel formation.

The strength of our approach is particularly evident in the documentation of the dust export from North Africa into the Eastern Mediterranean Sea. Clay mineral EM1 shows a sharp, strong increase at the termination of each AHP, immediately followed by a gradual decline. This decline reflects a decrease in the availability of fine-grained, kaolinite- and palygorskite-bearing sediments in northern Africa rather than changes in aridity. Grain size EM1 and XRF EM1 represent Saharan dust in the silt fraction and the bulk sediment, respectively. These end members document a continuously high dust export during the dry phases, while dust export was considerably reduced during all AHPs.

The loadings of clay mineral EM1 distinctly drop within sapropel S5, which is associated with the strongest humid phase in our record. This distinct decrease indicates an interruption of the dust influx. This signal is only apparent in the clay mineral data while the other dust end members show less pronounced decrease in their loadings compared to that of clay mineral EM1. This finding suggests that the dust input from North Africa during this humid period is strongly reduced but not completely interrupted.

Generally, fluvial sediment input into the Eastern Mediterranean Sea is controlled by the insolation-driven movement of the ITCZ and the resulting increase/decrease of humidity in the borderlands. Changes in the fluvial discharge to the Eastern Mediterranean Sea are strongly related to the Northern Hemisphere insolation with enhanced discharge during the insolation maxima and a strongly reduced discharge during the summer insolation minima. Clay mineral EM3 and grain size EM2 show a strong correlation with the Northern Hemisphere summer insolation, reflecting a higher humidity in North Africa and South Anatolia. We cannot distinguish between the suspension loads of the south Anatolian rivers and the Nile because of their similar geochemical and isotopic signatures. Clay mineral EM2 and XRF EM2 show high loadings during the humid periods and lower loadings during the drier phases but do not show a strong relation to orbital climate forcing. XRF EM2 most likely represents the riverine sediment influx from the Nile. The composition of clay mineral EM2 indicates that it likely reflects the riverine sediment input into the northern Aegean Sea, followed by transport to the Ionian Sea south of Crete via ocean currents. Grain size EM3 cannot be associated with a specific sediment provenance but most likely represents fluvial sediment as well.
